# SmartFlares fail to reflect their target transcripts levels

**DOI:** 10.1038/s41598-017-11067-6

**Published:** 2017-09-15

**Authors:** Maria Czarnek, Joanna Bereta

**Affiliations:** Faculty of Biochemistry, Biophysics and Biotechnology, Jagiellonian University in Kraków, Gronostajowa 7, 30-387 Kraków, Poland

## Abstract

SmartFlare probes have recently emerged as a promising tool for visualisation and quantification of specific RNAs in living cells. They are supposed to overcome the common drawbacks of current methods for RNA analysis: the need of cell fixation or lysis, or the requirements for genetic manipulations. In contrast to the traditional methods, SmartFlare probes are also presumed to provide information on RNA levels in single cells. Disappointingly, the results of our comprehensive study involving probes specific to five different transcripts, *HMOX1*, *IL6*, *PTGS2*, *Nrg1*, and *ERBB4*, deny the usefulness of SmartFlare probes for RNA analysis. We report a total lack of correlation between fluorescence intensities of SmartFlare probes and the levels of corresponding RNAs assessed by RT-qPCR. To ensure strong differences in the levels of analysed RNAs, their expression was modified via: (*i*) *HMOX1*-knockdown generated by CRISPR-Cas9 genome editing, (*ii*) hemin-mediated stimulation of *HMOX1*- and IL1β-mediated stimulation of *IL6*- and *PTGS2* transcription, (*iii*) lentiviral vector-mediated *Nrg1* overexpression. Additionally, *ERBB4*-specific SmartFlare probe failed to distinguish between *ERBB4*-expressing and non-expressing cell lines. Finally, we demonstrated that fluorescence intensity of *HMOX1*-specific SmartFlare probe corresponds to the efficacy of its uptake and/or accumulation.

## Introduction

The majority of quantitative analyses of expression of specific RNAs require cell fixation or lysis to isolate RNA; consequently the cells are lost for further experiments. Moreover, most of the procedures provide only information on average expression levels of individual genes in a given cell population. Therefore, a method that enables detection of a specific RNA in single living cells would be highly desirable. The most popular existing method for RNA visualization inside living cells requires modification of the chosen transcript to contain multiple copies of MS2 bacteriophage stem-loop motif and the introduction of MS2 coat protein (MCP) fused to a fluorescent protein into the cells^1^. Because of the necessity of transcript tagging, this method is not applicable for the analysis of localization of endogenous transcripts and regulation of their expression. Lately, CRISPR-Cas9 system was successfully repurposed to allow endogenous RNA tracking^2^. However, none of the aforementioned methods enable to sort the cells that exhibit the desired expression profile. Recently, a novel tool for RNA detection in living cells, namely SmartFlare, has been brought onto the market. It is based on previously developed Nanoflare technology^3^. In brief, SmartFlare probes are ~13 nm gold nanoparticle-coupled single-stranded DNA (ssDNA) oligonucleotides designed to selectively bind a desired transcript. The oligonucleotides are hybridized to shorter ssDNA containing a fluorophore (Cy3 or Cy5). When a short oligonucleotide (called reporter strand) is bound to the longer one (called capture strand), fluorescence is quenched by the gold nanoparticle present in a close proximity to the fluorophore. However, when the target RNA is present, the short strand should be displaced and fluorescence is no longer quenched. The fluorescence intensity should correlate with the transcript level. Two additional probe types are available: scramble and uptake controls. The scramble probe is aimed at determining background fluorescence, as its capture strand is not complementary to any known transcript in human, mouse and rat. The uptake probe is a single oligonucleotide bound to a gold nanoparticle. This oligonucleotide is fluorescently labelled at a distal end, hence it is not quenched by the gold nanoparticle. The signal of the uptake probe, which is constantly fluorescent and is not sequence specific, should verify the ability of the cells to engulf the probes. According to the manufacturer, SmartFlare probes enable both live cell imaging as well as flow cytometry analysis and cell sorting.

Whenever a new technique emerges, rigorous and detailed tests are needed to avoid data misinterpretation. Here, we present a comprehensive evaluation of SmartFlare usefulness to analyse specific transcript levels in living cells. We took diverse approaches to alter certain mRNAs expression: (*i*) generation of *HMOX1*-knockdown cells by CRISPR-Cas9-mediated genome engineering, (*ii*) stimulation of gene expression with classical stimulators (hemin for *HMOX1* or IL1β for *IL6* and *PTGS2*), and (*iii*) transduction of cells with vectors coding for mouse neuregulin 1 (NRG1). Then, we compared SmartFlare HMOX1-Cy5, IL6-Cy5, PTGS2-Cy5, and Nrg1-Cy5 fluorescence intensities with the levels of corresponding mRNAs evaluated by RT-qPCR. We also measured ERBB4-Cy5 fluorescence signals in various cell lines and compared them with *ERBB4* expression profile obtained by RT-PCR.

We show that HMOX1-Cy5, IL6-Cy5, PTGS2-Cy5 and Nrg1-Cy5 SmartFlare fluorescence intensities do not correlate with mRNA levels measured by RT-qPCR. Using cell lines devoid of *ERBB4* we prove that ERBB4-Cy5 SmartFlare probe is not able to selectively label *ERBB4*-expressing cells. Finally, we provide evidence that within the same cell type, SmartFlare fluorescence intensity is associated with the ability of the cells to internalize/accumulate SmartFlare probes.

## Results

### SmartFlare probes do not discriminate between cells that strongly differ in specific transcript levels

We planned to use a SmartFlare probe to sort the population of 293T cells that became *HMOX1*-deficient as a result of CRISPR-Cas9-mediated genome editing. 293T cells were transiently transfected with *HMOX1-*targeting pX330-Pac-Cer vector coding for human codon-optimized Cas9 and human *HMOX1-*specific sgRNA or with control empty pX330-Pac-Cer. Unlike control cells, the cells transfected with *HMOX1-*targeting pX330-Pac-Cer showed highly efficient introduction of mutations in *HMOX1* locus assessed by CELI mismatch detection assay (Supplementary Figure S1). We expected that *HMOX1*-targeted 293T cells would contain a subpopulation, in which indel mutations had caused a frameshift in all loci. It should result in strongly diminished *HMOX1* mRNA level due to degradation of transcripts containing premature stop codons. However, we were not able to pinpoint this population using *HMOX1*-specific SmartFlare probe (data not shown).

**Figure 1:**
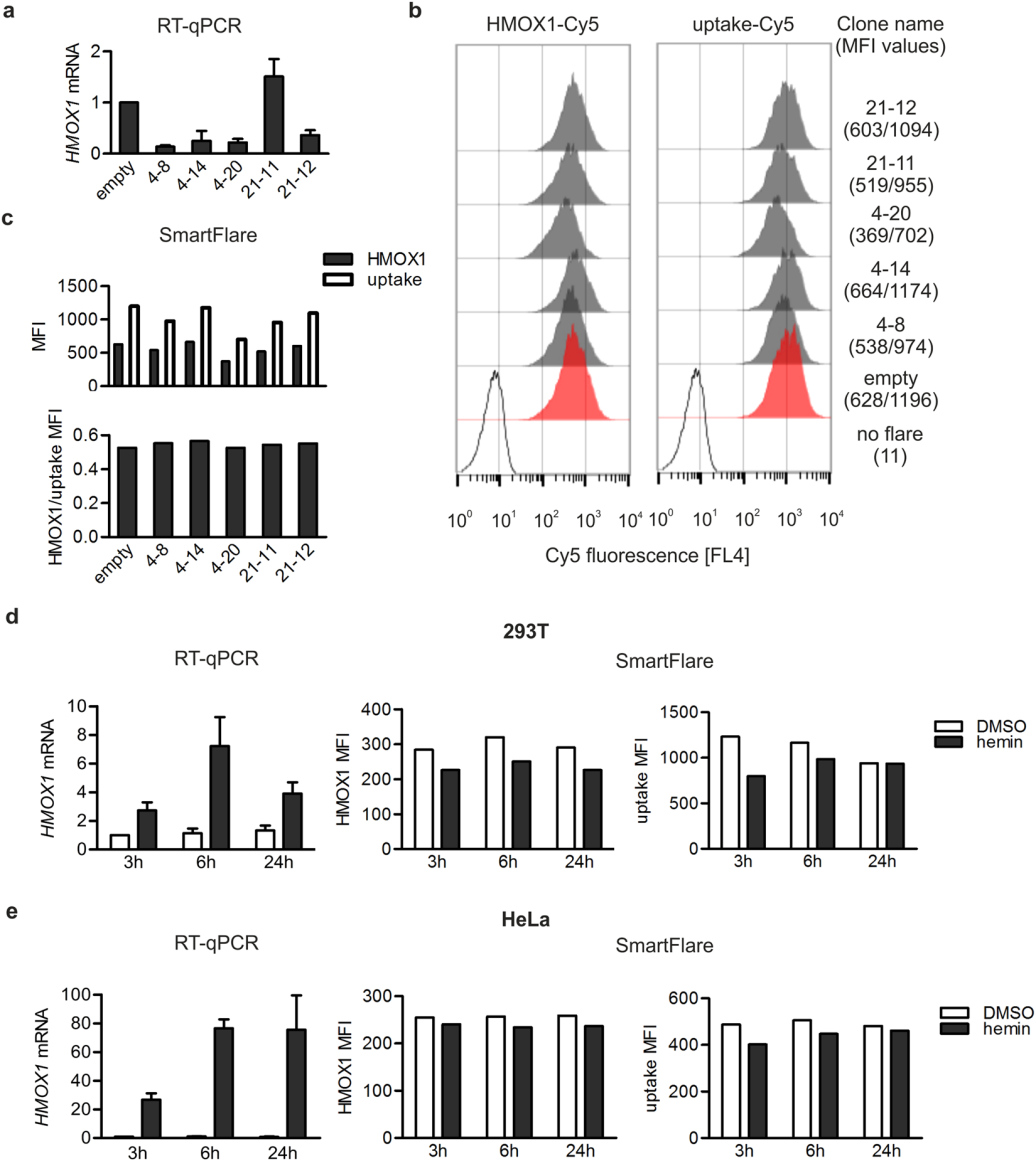
Assessment of *HMOX1* transcript level by RT-qPCR and SmartFlare probe. (**a**) RT-qPCR analysis of *HMOX1* expression in control (transfected with empty plasmid) and *HMOX1*-knockdown 293T cell clones generated by CRISPR-Cas9 genome engineering. Values represent the mean ± SEM from four independent experiments performed in duplicates. (**b**,**c**) Flow cytometry analysis of HMOX1-Cy5 and uptake-Cy5 fluorescence in control and *HMOX1*-knockdown 293T cell clones. The MFI values of HMOX1-Cy5 and uptake-Cy5 are shown in parentheses. The MFI of HMOX1-Cy5 was divided by that of uptake-Cy5 and resulted value is shown as HMOX1/uptake MFI. Representative results of 3 independent experiments are shown. (**d**,**e**) RT-qPCR and SmartFlare flow cytometry analysis of *HMOX1* expression in 293T (**d**) or HeLa cells (**e**) incubated with DMSO (solvent) or hemin for indicated time periods. In graphs showing RT-qPCR results, values represent the mean ± SEM from 3 independent experiments performed in duplicates. Flow cytometry analyses representative of 3 independent experiments performed in parallel with RT-qPCR for both cell lines are shown.

Therefore, to generate cells with more discrete and uniform *HMOX1* transcript levels rather than a heterogeneous population of wild-type, partial knockout, and knockout cells, 293T cells were subjected to single cell cloning. A fraction of single-cell-derived clones harbouring a mutation in *HMOX1* locus showed *HMOX1* expression reduced by 64–86% as measured by RT-qPCR (Fig. 1a). We also isolated a 293T cell clone with considerably higher *HMOX1* mRNA level compared with *HMOX1* level in cells transfected with a control vector (Fig. 1a, clone 21–11). Surprisingly, despite significant differences in *HMOX1* transcript levels between isolated cell clones and control cells, there was no parallel difference in HMOX1-Cy5 SmartFlare fluorescence level evaluated by flow cytometry (Fig. 1b,c). In clone 4–20 diminished *HMOX1*-specific SmartFlare signal was apparently accompanied by decreased fluorescence of the uptake probe (further referred to as uptake-Cy5), therefore we decided to normalize HMOX1-Cy5 fluorescence signal to that of uptake-Cy5 by dividing mean fluorescence intensity (MFI) of HMOX1-Cy5 by MFI of uptake-Cy5 (Fig. 1c). The HMOX1/uptake ratios were almost identical across all *HMOX1*-knockdown and control 293T cells, suggesting that all differences in HMOX1-Cy5 fluorescence between cell populations reflected differences in the probe uptake rather than in *HMOX1* transcript levels.

The disparity between RT-qPCR and HMOX1-Cy5 fluorescence could possibly be explained by low basal *HMOX1* transcript levels in 293T, possibly below SmartFlare resolution threshold. To verify this hypothesis, we stimulated wild type 293T cells with hemin, a known activator of *HMOX1* transcription, and examined SmartFlare fluorescence using flow cytometry. Although *HMOX1* levels increased about three- and seven times in 293T cells treated with hemin for 3 or 6 h respectively, we did not detect any changes in SmartFlare fluorescence intensity (Fig. 1d). Even more dramatic effect of hemin was observed in HeLa cells (Fig. 1e). However, even as high as 77-fold increase in *HMOX1* transcript level was not accompanied by any changes in *HMOX1*-specific SmartFlare probe fluorescence (Fig. 1e). We concluded that *HMOX1*-specific SmartFlare probe does not discriminate between cells expressing high, normal, and negligible *HMOX1* levels.

Another possible explanation for the lack of correlation between SmartFlare fluorescence and *HMOX1* transcript levels could be that a degraded probe was used, in which fluorescence was already unquenched before its application to the cells. However, a strong increase in the fluorescence of *HMOX1*-specific SmartFlare observed in response to DTT, which is known to detach probes from gold nanoparticles^4^, ruled out this possibility. The effect of DTT on HMOX1-Cy5 and three other probes used in this study (described below) is shown in Supplementary Figure S2.

**Figure 2:**
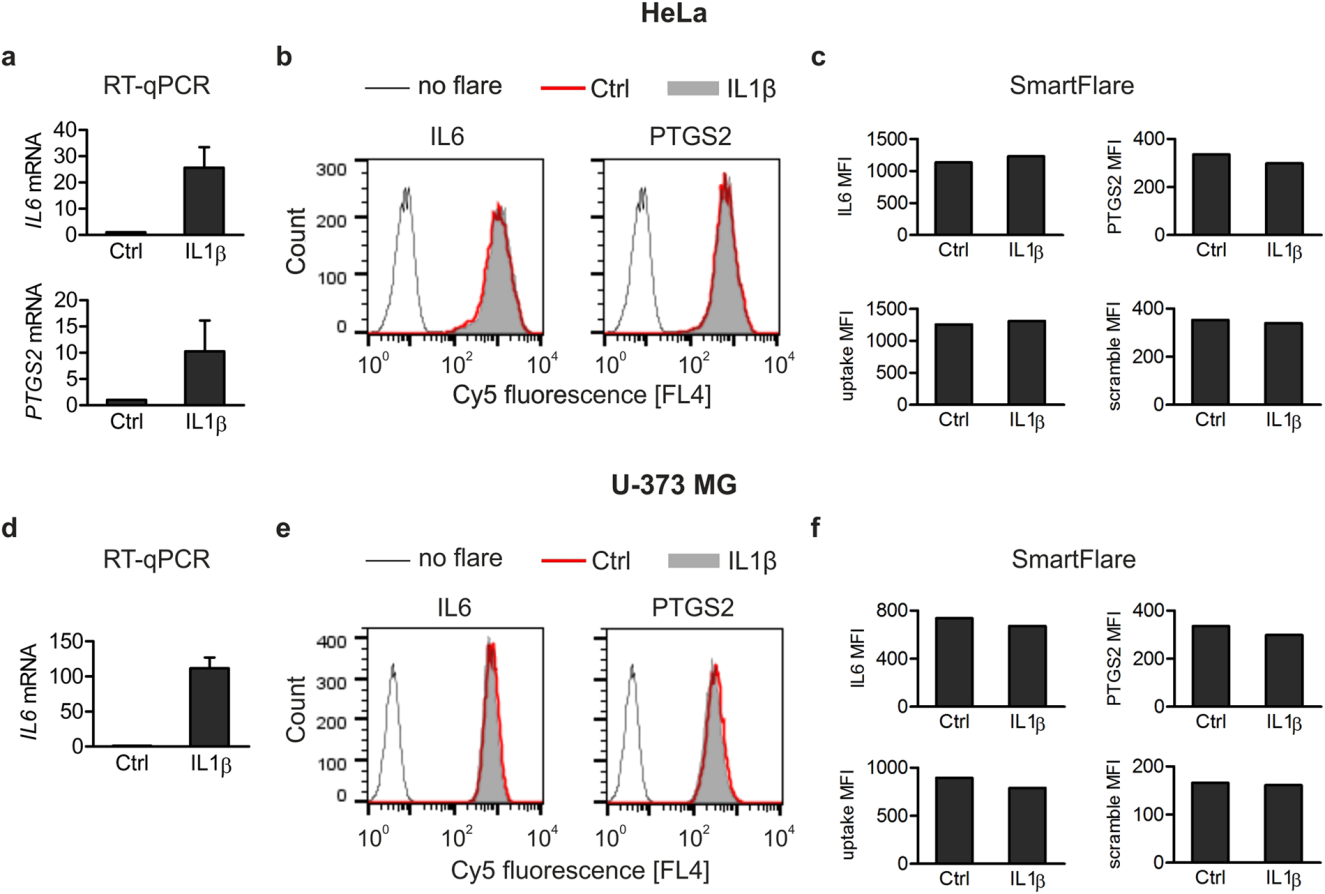
Analysis of *IL6* and *PTGS2* transcript levels by RT-qPCR and SmartFlare probes. (**a**,**d**) RT-qPCR analysis of *IL6* and *PTGS2* mRNA in control and IL1β-treated HeLa (**a**) and U-373 MG (**d**) cell lines. Values represent the mean ± SEM from 3 independent experiments performed in duplicates. (**b**,**e**) Flow cytometry histograms of cell-associated IL6-Cy5, PTGS2-Cy5 and uptake-Cy5 fluorescence in control and IL1β-stimulated HeLa (**b**) and U-373 MG (**e**) cells. (**c**,**f**) The MFI of IL6-Cy5, PTGS2-Cy5, uptake-Cy5 and scramble-Cy5 from data shown in (**b**) and (**e**). Shown are representative results of 3 independent experiments.

To verify whether or not *HMOX1* represents a single case we investigated applicability of SmartFlares for analysis of expression of a few other transcripts. First, we examined whether SmartFlare signals reflect changes in the expression levels of inflammatory mediators, interleukin 6 (*IL6*) and prostaglandin-endoperoxidase synthase-2 (*PTGS2*, also known as cyclooxygenase-2), occurring in response to major proinflammatory cytokine, IL1β. As expected, RT-qPCR analysis revealed strong (10 to 100-fold) increase in *IL6* and *PTGS2* mRNA levels in IL1β-treated HeLa and U-373 MG cells in comparison with untreated counterparts (Fig. 2a,d; data concerning *PTGS2* in U-373 MG are not presented because, unlike in IL1β-stimulated cells, in control cells *PTGS2* mRNA was undetectable). However, these substantial differences in *IL6* and *PTGS2* levels were not accompanied by any changes neither in *IL6*- nor in *PTGS2*-specific SmartFlare MFIs assessed by flow cytometry (Fig. 2b,c,e,f). To address the question whether SmartFlare probes may affect IL1β-mediated changes in the expression of analysed transcripts, we measured *IL6* and *PTGS2* mRNA levels in HeLa and U-373 MG cells preincubated overnight (according to manufacturer’s instruction) with 100 pM *PTGS2*-specific probe and then stimulated with IL1β for 3 h. The results showed that in HeLa cells the SmartFlare did not influence IL1β-induced upregulation of *IL6* and *PTGS2* expression, however it slightly, by about 25%, diminished *IL6* expression in IL1β-stimulated U-373 MG cells (Supplementary Figure S3). We did not test whether this difference was due to the presence of *PTGS2*-specific sequence or simply of gold nanoparticles; however, a potential influence of SmartFlare on analysed transcripts levels should be taken into consideration.

We also investigated the usefulness of SmartFlare probes for quantification of transcripts overexpressed due to transduction of cells with lentiviral vectors. In our model, MC38CEA cells, which express endogenous NRG1, were transduced with lentiviral vectors coding for mouse NRG1 type I or NRG1 type III, or with empty vector. Type I and type III NRG1 are products of the same gene and *Nrg1*-specific SmartFlare probe used in this study should recognize both transcripts.

**Figure 3:**
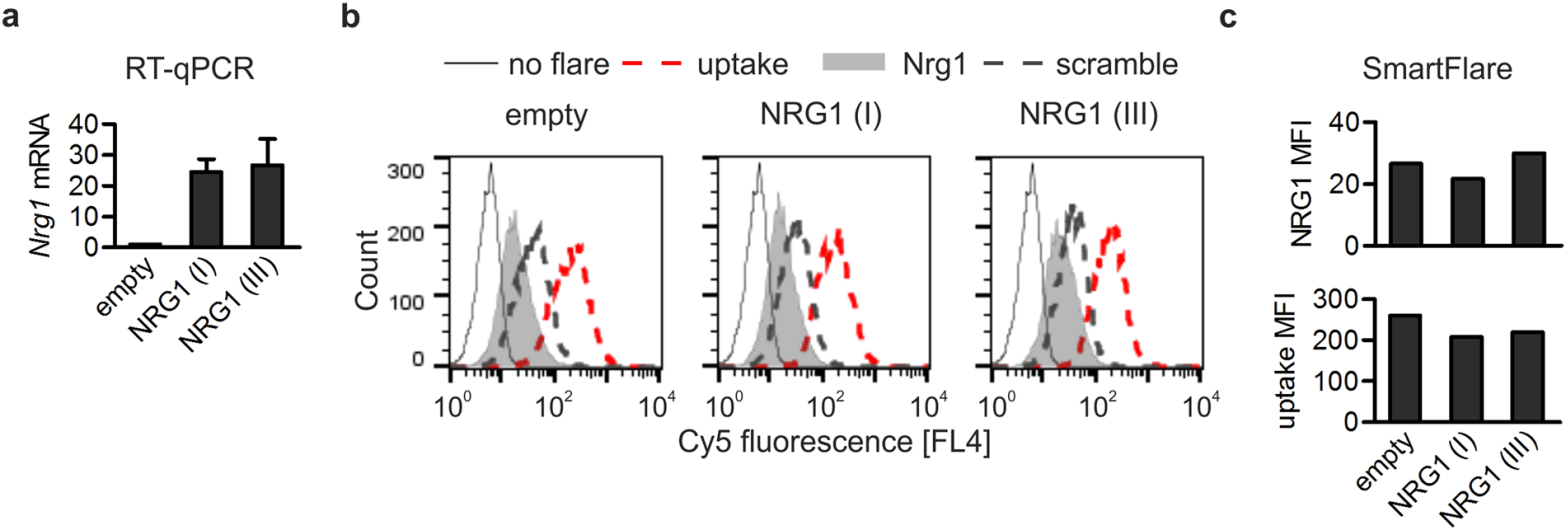
Analysis of *Nrg1* transcript level by RT-qPCR and SmartFlare probe in MC38CEA cells transduced with lentiviral vectors. (**a**) RT-qPCR analysis of *Nrg1* mRNA level in MC38CEA cells transduced with control, empty vector (empty), vector coding for murine NRG1 type I (NRG1 (I)) or NRG1 type III (NRG1 (III)). Values represent the mean ± SEM from 3 independent experiments performed in duplicates. (**b**) Representative flow cytometry histograms of Nrg1-Cy5, uptake-Cy5 and scramble-Cy5 fluorescence. (**c**) The MFI values of Nrg1-Cy5 and uptake-Cy5 from data shown in (**b**). Flow cytometry analyses representative of 3 independent experiments performed in parallel with RT-qPCR are shown.

Transduction of the cells with vectors encoding NRG1 types I or III resulted, respectively, in 24-fold and 27-fold upregulation of *Nrg1* expression as evaluated by RT-qPCR (Fig. 3a). Despite the evident differences in *Nrg1* mRNA levels, MFI values of SmartFlare probe analysed by flow cytometry did not show any significant differences between cells with moderate and high *Nrg1* mRNA levels (Fig. 3b,c). Moreover, MFI of *Nrg1*-specific probe was comparable to that of a scramble probe in all MC38CEA populations, although *Nrg1* transcript was abundant (Fig. 3b and Supplementary Table S1). MC38CEA cells were able to internalize the probes since the uptake probe MFI reached values that were substantially higher than MFI in control, unflared cells (Fig. 3b). Thus, also in the case of *Nrg1*, SmartFlare technique failed to reflect substantial differences in the transcript levels (Fig. 3c).

### *ERBB4*-specific SmartFlare probe fails to discriminate between *ERBB4-*expressing- and nonexpressing cell lines

**Figure 4:**
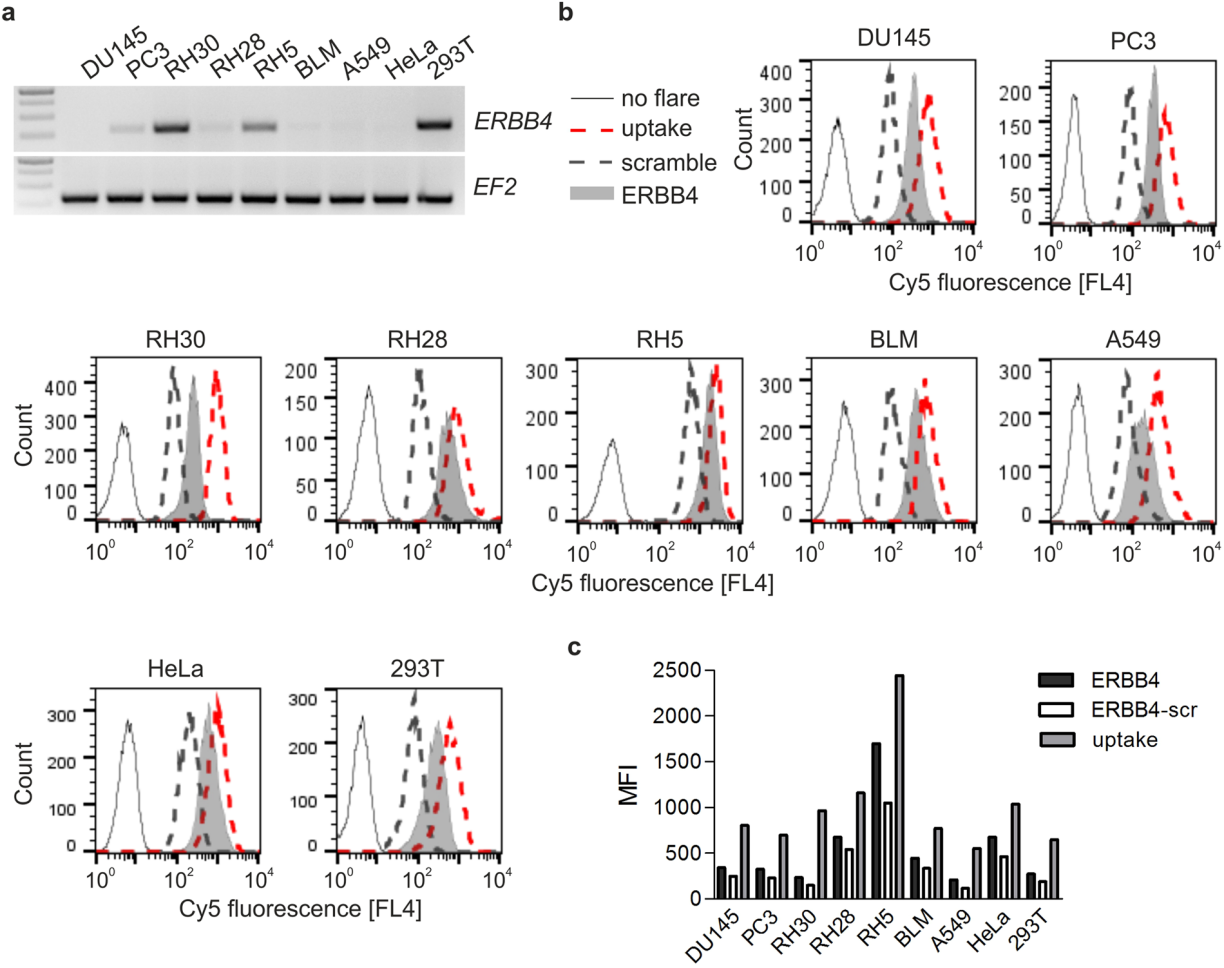
Detection of *ERBB4* transcript in multiple cell lines by RT-PCR and SmartFlare probe. (**a**) RT-PCR analysis of the expression of *ERBB4* in various human cell lines. *EF2* serves as a control of the samples quality. Representative result coming from three independent RNA isolations. The image (negative of original) presents two parts of the same gel. Exposition time was set to “autoexposition”, separately for *ERBB4* and *EF2*. The original images are to be found in Supplementary Information as Supplementary Figure S7. (**b**) Representative flow cytometry histograms of ERBB4-Cy5, uptake-Cy5 and scramble-Cy5 fluorescence. (**c**) The MFI of ERBB4-Cy5 without scramble-Cy5 MFI subtraction (denoted as “ERBB4”) and with scramble-Cy5 MFI subtraction (“ERBB4 - scr”) and uptake-Cy5 derived from data shown in (**b**). Flow cytometry analyses representative of 3 independent experiments are shown.

SmartFlare probes are considered a useful tool for analysis of a gene of interest expression profile across different cell lines or cell subpopulations. We thus tested a number of human cell lines in respect of yet another transcript expression, namely *ERBB4*, using RT-PCR and compared the results with *ERBB4*-specific SmartFlare fluorescence signals. *ERBB4* mRNA was detected in 293T, RH5 and RH30; weak band corresponding to *ERBB4* transcript was also observed in PC3 and RH28 cells (Fig. 4a). *ERBB4* mRNA was undetectable in HeLa, A549, BLM and DU145 cells. Discordantly, in all cell lines tested, *ERBB4-*specific probe signals exhibited higher MFI than that of the scramble probe (Fig. 4b), which is considered indicative of the transcript presence^5–7^. When MFI of ERBB4-Cy5 in 293T (274) is compared with that of RH28 (675), one may infer that RH28 cells express higher level of *ERBB4* than 293T, which is contradicted by the RT-PCR results. Unlike in our previous experiments performed on single cell lines, in this experiment, carried out across various cell lines, the fluorescence signals of both scramble and uptake probes differed significantly among the cell lines. Nevertheless, subtracting the scramble probe MFI values from *ERBB4*-specific MFI values did not change the picture (Fig. 4c). MFI values calculated in this way still failed to reflect differences in *ERBB4* levels revealed by RT-PCR (Fig. 4c).

### Cell-associated fluorescence of HMOX1-Cy5 SmartFlare reflects efficiency of its uptake rather than the transcript level

All our experiments indicated that even if cells strongly differ in particular transcript levels, they cannot be distinguished by SmartFlare probes. The results suggested that the differences in the intensities of intracellular SmartFlares fluorescence, rather than be related to the mRNA levels, may in fact reflect unequal abilities of the cells to accumulate or preserve the probes.

**Figure 5:**
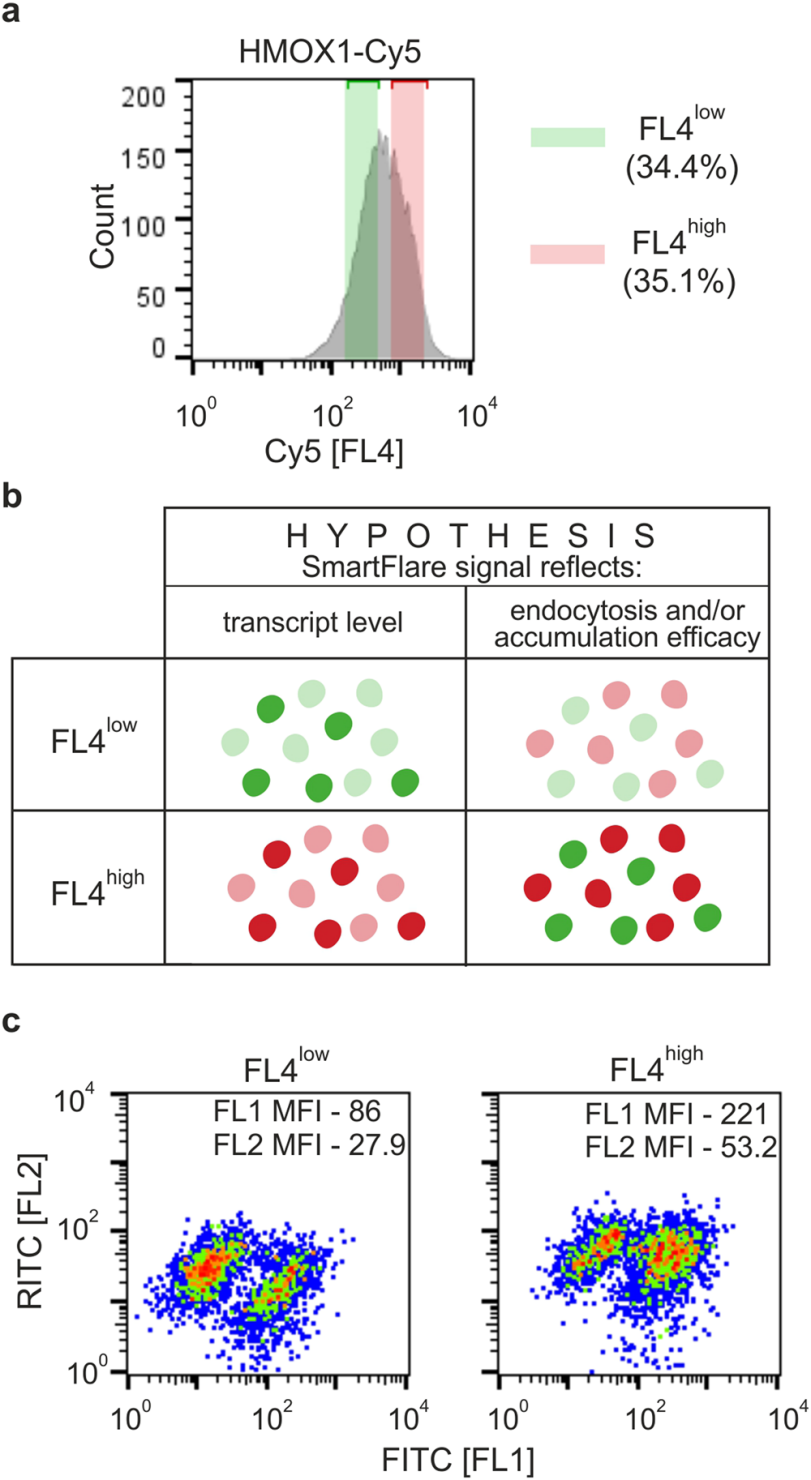
Flow cytometry analysis of HMOX1-Cy5 SmartFlare fluorescence in 4–8 and 21–11 293T cell clones preloaded with fluorescently labelled dextrans. (**a**) The cell clones 4–8 (showing negligible *HMOX1* expression) and 21–11 (showing high *HMOX1* expression) were preloaded with FITC- or RITC-dextran, respectively, then mixed and incubated overnight with HMOX1-Cy5 SmartFlare probe. (**b**) The diagram presents the idealized hypothetical results of the experiment verifying the cause of differences in SmartFlare signal intensities. Green ovals represent 4–8 cells loaded with FITC-dextran, while red ovals represent 21–11 cells loaded with RITC-dextran. (**c**) Two subpopulations of the cells were gated based on Cy5 fluorescence intensity: FL4^low^ and FL4^high^ (left panel). Cell-associated FITC and RITC fluorescence in FL4^low^ and FL4^high^ subpopulations are shown as dot plots of FITC [FL1] vs RITC [FL2] (right panel). Representative result of 2 independent experiments is shown.

To verify this hypothesis we loaded 293T clone 4–8, which showed strongly diminished *HMOX1* expression compared to control cells (Fig. 1a), with FITC-dextran and clone 21–11, which showed increased *HMOX1* level to about 150% of that in control cells (Fig. 1a) with RITC-dextran. Then fluorescently labelled cells were mixed and incubated with *HMOX1-*specific SmartFlare probe. Two 293T subpopulations were gated based on Cy5 fluorescence intensity: FL4^low^ with MFI = 309 and FL4^high^ with MFI = 1142 (Fig. 5a). If SmartFlare fluorescence intensity correlated with *HMOX1* transcript level, green fluorescence (channel FL1) should prevail in FL4^low^ subpopulation due to low *HMOX1* mRNA level in FITC-labelled 4–8 clone; conversely, red fluorescence (channel FL2) should be predominant in FL4^high^ subpopulation as a consequence of enhanced *HMOX1* expression in clone 21–11 (Fig. 5b). Instead, we observed equal distribution of cells with green and red fluorescence in both FL4^low^ and FL4^high^ subpopulations (Fig. 5c). Moreover, the mean intensities of both green and red fluorescence were higher in FL4^high^ subpopulation than those in FL4^low^ cells. The same effect was observed when clone 4–8 was loaded with RITC-dextran and clone 21–11 with FITC-dextran (data not shown). Therefore, we believe that SmartFlare fluorescence intensity does not correspond to target mRNA levels, but most likely is associated with the efficiency of SmartFlare probe uptake and accumulation.

## Discussion

Most of our findings contradict the conclusions of a number of studies that utilized SmartFlare and Nanoflare probes to evaluate differences in mRNA levels in living cells^3, 8–12^. In contrast to those publications, we were not able to detect any correlation between SmartFlare probes fluorescence intensities and their target transcripts levels evaluated by RT-qPCR. The most apparent explanation would be that the expression levels of the analysed transcripts were below SmartFlare detection limit. However, in some cases, the amounts of the analysed transcripts were close to that of *EF2* (Supplementary Table S1), which belongs to highly expressed genes^13^. Nevertheless, the manufacturer claims that SmartFlare probes are able to detect mRNAs that are far less abundant (amplify in RT-qPCR “as late as cycle 34” according to SmartFlare User Guide, Literature Code TP5764EN00), and although this information is not precise it suggests high sensitivity of SmartFlare technique. In our experiments Cq of analysed transcripts ranged from about 19 to about 31, thus was above manufacturer’s stated detection limit.

In our opinion the vast majority of experiments employing SmartFlare probes are erroneously interpreted due to the lack of appropriate control, i.e. uptake control. This control is often not mentioned at all^3, 8–11^, or the authors claim that the fluorescence of the uptake probe was similar in all experimental groups without presenting the results^12^.

Our results support the hypothesis that target-specific SmartFlare fluorescence intensity does not depend on the target transcript level but rather on efficiency of the probe accumulation. To rule out this notion, one should unconditionally analyse the efficiency of cellular uptake using an adequate probe, i.e. a probe with fluorescence intensity in a range similar to that of a target-specific probe. The uptake probes conjugated with Cy3 or Cy5 fluorochrome are offered. However, the Cy3-based probes have some limitations. They are not suitable for analysis performed with common flow cytometers equipped only in basic lasers. Although Cy3 should be excited to about 50% of maximum with an argon laser, in our experiments MFI of Cy3-uptake control was only slightly higher than that of control, unflared cells (Supplementary Figure S4). Strong overlapping of uptake and 18S rRNA probes fluorescence and cell autofluorescence disqualifies such probes for normalisation of target-specific signals.

When a transcript level is to be compared between different cell types, another control should be included. In this type of experiments, it is essential to measure also MFI of a housekeeping transcript-specific SmartFlare, as MFI of a SmartFlare probe specific to a transcript of interest may differ in various cell types irrespective of the transcript content in these cells. The probes specific to a high-abundance transcripts (18S rRNA, β-actin, GAPDH), are currently available from SmartFlare manufacturer. The lack of proper uptake and/or housekeeping transcript controls makes us believe that sorting of mixed cell populations presented in Merck brochure (Cell sorting based on RNA detection in living cells using SmartFlare^TM^ RNA Detection Reagents, Literature Code AN4665EN00), is actually based on differences in efficacy of a SmartFlare probe uptake and/or retention and not on differences in the levels of targeted mRNA. Moreover, our results indicate that cell-associated SmartFlare fluorescence strongly correlates with the applied probe concentration (Supplementary Figure S5) and in consequence, with the amount of the internalized probe.

According to the manufacturer’s information and a general opinion presented in publications, the MFI of target-specific SmartFlare probe that is higher than MFI of a scramble probe is regarded as an evidence of the transcript presence^5–7^. However, our observations contradict this notion, as the fluorescent signal of *Nrg1*-targeting probe is comparable to that of a scramble probe despite the abundance of *Nrg1* transcript and strong ERBB4-Cy5 fluorescent signal, substantially higher than that of a scramble probe, is observed in cells that lack *ERBB4* expression. This once again underlines that SmartFlare fluorescence does not correlate with targeted RNA levels.

One can argue that if our hypothesis is true, i.e. the fluorescence intensity of the SmartFlares reflects the efficiency of their uptake/accumulation and not RNA abundance, then the fluorescence signals of the scramble and specific probes should be similar. It would be true, provided that the probes were labelled to the same extent. As uneven labelling of the probes denies the principle of SmartFlare methodology, it did not cross our minds to routinely evaluate their labelling levels. To completely dispel any doubts concerning this issue, we performed additional analysis of a freshly purchased pair of SmartFlares: scramble-Cy5 and β-actin-Cy5. We chose probe specific to β-actin because at the time of the analysis any probe used in this study was no longer available. The measurements of fluorescent signals of the probes subjected to DTT-mediated unquenching indicated that the probes were not evenly labelled. The signal derived from the specific probe was twice as high as that of the scramble control (Supplementary Figure S6). We observed the same phenomenon of uneven labelling of the scramble and gene-specific probes for Cy3-attached probes (data not shown). This may explain the observed differences in fluorescence signals between scramble- and specific probes, otherwise puzzling in the light of our hypothesis.

A recent works support our belief that SmartFlare fluorescence does not depend on a specific transcript level. Using electron microscopy, groups of Lévy and Aurich independently demonstrated that SmartFlare probes are trapped inside intracellular vesicles and do not have a chance to interact with their target mRNAs^14, 15^. Thus, most probably the manufacturer of SmartFlare probes did not overcome the long-lasting problem concerning cytoplasmic delivery of RNA-targeting probes^16^. The use of oligonucleotide molecular beacons that recognize specific mRNAs was proposed already in 1998 by Sokol *et al*. However, to avoid strong background signal associated with endosome-trapped probes, they microinjected the beacons directly to the cytoplasm^17^. Curiously, the group of Mirkin (the same group, which developed Nanoflare probes) confirmed prevalent vesicular localization of spherical nucleic acid (SNA)^18^. As they claim, the fluorescent signal detected in cells, may arise from lysosomal DNAse II digestion of SNA and release of the fluorophore^18^. If it was the case, it would immediately disqualify SNA-based SmartFlares as an analytical tool. However, our preliminary results may suggest that a failure of SmartFlares does not depend on their lysosomal degradation, because cell treatment with moderate concentration of chloroquine that prevents endosome acidification, has minor effect of cellular fluorescence intensities of SmartFlares (data not shown). The only exception is RAW264.7 cell line, in which chloroquine treatment leads to noticeable increase in SmartFlare cellular fluorescence (data not shown).

Is it possible to reconcile the previous results of SmartFlare-based experiments with our hypothesis? In our opinion, yes. We believe that in some cases, higher efficiency of internalization/accumulation of nanoparticles (including SmartFlares) may occur in parallel with other cell features, such as higher expression of anti-apoptotic and promigratory genes and, as a result, more aggressive phenotype of cancer cells. In this scenario, cells with high fluorescence of SmartFlare probe (due to high internalization rate) will also display higher expression of cancer-related genes than cells with low SmartFlare fluorescence. Thus, correlation between intensity of SmartFlare fluorescence and gene expression level would be entirely coincidental, as we believe is in the case of sorting of melanoma^10^ and prostate cancer cells^11^ based on fluorescence intensity of NODAL and AMACR SmartFlares, respectively. Similarly, higher internalization efficiency of *NANOG*-specific probe by so-called cancer stem cells (CSC) compared to cancer non-SC might explain successful sorting of the former population^6^. As a matter of fact, the potential differences in endocytosis efficacy between various subpopulations of cancer cells have not yet been studied; the only support for this notion comes from the observation by Yucesoy that incubation of cancer cells with 20 nm gold particles for 6 h resulted in higher accumulation of these nanoparticles in CSC than in the cells with a non-stem phenotype^19^. Interestingly, gold nanoparticles by themselves may enhance tumour cell invasion as reported for lung cancer cell lines treated with 20 nm gold nanoparticles^20^. SmartFlare probes specific for transcripts of pluripotency markers NANOG and GDF3 were also used to select induced pluripotent stem cells^21^. Interestingly, preliminary experiments performed on mouse V6.5 embryonic stem cells showed that fluorescence-activated cell sorting using *Nanog-* or *Gdf3-*specific SmartFlares resulted in the subpopulations that did not differ (*Gdf3*) or did not significantly differ (*Nanog*) in the transcript levels as determined by RT-qPCR^21^. This observation, although not commented by the authors, are in agreement with our hypothesis.

Based on our results, we conclude that: (*i*) SmartFlare probes do not provide information on a particular transcript presence or absence; (*ii*) SmartFlare probes cannot distinguish the cells with high level from the cells with low level of a given mRNA; (*iii*) fluorescence intensity of a SmartFlare probe may be correlated with efficiency of the probe uptake; (*iv*) SmartFlare fluorescence intensity linearly correlate with the applied probe concentration.

We would also like to make use of this publication to appeal to the research community to provide, along with each new method and new research tool, a detailed protocol that explains the significance of all required controls as well as a guideline for the authors on minimal information that should be provided to allow reliable interpretation and critical evaluation of presented results.

## Materials and Methods

### Cell lines

293T (human embryonic kidney cells, ATCC CRL-3216), U-373 MG (Uppsala) (human astrocytoma cells, ECACC 08061901), PC3 (human prostate cancer cells, ATCC CRL-1435), DU145 (human prostate cancer cells, ATCC HTB-81) and MC38CEA (murine colon cancer cells expressing human carcinoembryonic antigen^22^ were cultured in DMEM (Lonza), HeLa (human cervical cancer cells, ATCC CCL-2) in EMEM (Lonza), A549 (human lung cancer cells, ATCC CCL-185) in F12 (Lonza), and BLM (human melanoma cells^23^, RH5, RH28 and RH30 (human rhabdomyosarcoma cell lines^24^, a gift from Dr. Peter Houghton, Greehey Children’s Cancer Research Institute) in RPMI 1640 (Lonza). All media were supplemented with 10% heat-inactivated fetal bovine serum (BioWest).

### Plasmid construction

sgRNA targeting human *HMOX1* gene was designed using CRISPR Design Tool (crispr.mit.edu). pX330-U6-Chimeric_BB-Cbh-hSpCas9 plasmid^25^, a gift from Feng Zhang (Addgene plasmid # 42230), was modified to express SpCas9, puromycin N-acetyltransferase (Pac) and Cerulean fluorescent protein (Cer) from a single promoter by inserting T2A and P2A coding sequences between SpCas9, Pac and Cer coding sequences (detailed cloning strategy is described in Supplementary Matherials and Methods and Supplementary Table S2). The resulted plasmid is hereinafter referred to as pX330-Pac-Cer. Phosphorylated and annealed oligonucleotides coding for *HMOX1*-targeting portion of the sgRNA were cloned into *Bbs*I-digested and dephosphorylated pX330-Pac-Cer. Oligonucleotide sequences are listed in Supplementary Table S3.

cDNAs coding for mouse NRG1 type I and NRG1 type III were PCR-amplified from reverse-transcribed poly(A)^+^ fraction of RNA isolated from the brain of 3-week old C57BL/6 mouse (brain tissue was obtained from the animal house of the Faculty). RT and PCR were performed using ImProm-II Reverse Transcription System (Promega) and KAPA HiFi polymerase (KAPA Biosystems) with following primers: NRG1(I)_For: ATGTCTGAGCGCAAAGAAGG; NRG1(III)_For: ATGGAGATTTATCCCCCAGAC; NRG1_Rev: TTATACAGCAATAGGGTCTTGGT (for both NRG1 types I and III). PCR products were cloned into pJET1.2 plasmid using CloneJET PCR cloning kit (Thermo Scientific) and then subcloned into pLVX-IRES-puro vector (Clontech) using *Xho*I/*Xba*I restriction sites.

### Lentiviral vectors production and cell transduction

Two days before transfection 1 × 10^6^ 293T cells were plated in a 10-cm dish. 293T cells were transfected with 10.5 µg of DNA: 6 µg of pLVX expression constructs, 3 µg of 2^nd^ generation packaging plasmid psPAX2 and 1.5 µg of envelope plasmid pMD2.G (both were gifts from Didier Trono, Addgene plasmids #12260 and #12259, respectively) using Polyethylenimine HCl MAX, Linear, MW 40 000 (PEI; PolySciences) at a ratio of DNA to PEI 1:2. Media were renewed every 24 h for the next 3 days. Lentiviral vectors-containing media were pooled, filtered through a 0.45 μm filter and concentrated by centrifugation for 3 h at 23 000 g. Pseudoviral pellets were resuspended in 300 μl of serum-free DMEM; aliquots were stored at −80 °C. Pseudoviral stocks were titrated based on a concentration of viral-associated p24 protein using QuickTiter Lentivirus Titer Kit (Cell Biolabs). MC38CEA cells were transduced with 2000 lentiviral particles per cell (equivalent of MOI 10 as determined by flow cytometry analysis of MC38CEA cells transduced with known amount of lentiviral particles containing fluorescent marker ZsGreen). MC38CEA cells successfully transduced with pLVX-IRES-puro vectors were selected using puromycin (Bioshop, final concentration 5 μg/ml).

### Transfection and generation of *HMOX1-*knockdown 293T cells

293T cells (1 × 10^5^) were plated in 24-well plates one day before transfection. The cells were transfected with 500 ng of *HMOX1*-targeting pX330-Pac-Cer plasmid using jetPRIME reagent (Polyplus Transfection) according to the manufacturer’s protocol. Puromycin (BioShop, final concentration 10 µg/ml) was added 24 h after transfection and selective pressure was maintained for the next 48 h. Thereafter, the cells were seeded into 96-well plates, on average one cell per well, in order to obtain single cell-derived clones. Genomic DNA from the cell clones was extracted using Blood/Cell DNA Mini Kit (Syngen) following the manufacturer’s protocol and subjected to a mismatch detection assay with CELI nuclease (detailed description is available in Supplementary Information). The cell clones with mutation within *HMOX1* locus were subjected to RT-qPCR analysis of *HMOX1* expression and those with the lowest level of *HMOX1* transcript in comparison with the cells transfected with a control empty vector were selected for further experiments.

### Cell stimulation

Hemin (Calbiochem) was dissolved in DMSO. Human recombinant IL1β (R&D Systems) was reconstituted in DMEM. Hemin (final concentration of 10 µM) or equal volume of DMSO was added to 293T and HeLa cell cultures 3, 6 or 24 h before RNA isolation or flow cytometry analysis. IL1β (final concentration of 10 ng/ml) was added to HeLa and U-373 MG cultures 3 h before RNA isolation or flow cytometry analysis.

### RNA isolation, reverse transcription, RT-PCR and RT-qPCR

RNA was isolated from non-transduced cells by Chomczynski and Sacchi’s method^26^ and from the cells transduced with lentiviral vectors using DirectZol MiniPrep (ZymoResearch) with an in-column DNase I digestion step to avoid PCR amplification of genomically integrated *Nrg1* cDNA.

RNA concentration was determined with ND-1000 spectrophotometer (NanoDrop Technologies). Equal amounts of RNA samples (1 µg) were reverse-transcribed using M-MLV reverse transcriptase (Promega) and oligo(dT)_15_ primer following manufacturer’s recommendations. RT-PCR was performed with *Taq* polymerase (KAPA Biosystems) for 40 cycles under the reaction conditions recommended by the manufacturer, using 1 μl of RT reaction per 20 μl of PCR reaction volume. RT-PCR products were resolved in 2% agarose gel containing SERVA DNA Stain G (Serva Electrophoresis GmbH) in TAE buffer. DNA bands were visualized using gel imaging system Quantum ST5 (Vilber Lourmat); the color of the images was inversed using Fusion Capt Advance Fx5 program (Vilber Lourmat). The acquired images were not processed in any other way. The original images are included in Supplementary Information (Supplementary Figure S7).

RT-qPCR was performed on Eco Real-Time PCR System (Illumina) using KAPA SYBR Fast qPCR Master Mix (KAPA Bioscience) in 10 μl reaction volume. Each sample was assayed in duplicate. Specific amplification was confirmed by analysis of the melting curves and agarose gel electrophoresis of PCR products. EF2 (human and mouse) and HPRT (mouse) were used as reference genes. The fold change in expression was calculated using REST2009 software^27^; calculations included PCR efficiency correction. Primers used for RT-PCR and RT-qPCR are listed in Supplementary Table S4.

### Flow cytometry

SmartFlare probes recognizing human *HMOX1*, *PTGS2*, *IL6*, *ERBB4* and mouse *Nrg1* transcripts as well as uptake- and scramble control probes, all labelled with Cy5 dye, were purchased from Merck (catalog numbers respectively: SF-1171, SF-1818, SF-456, SF-416, SF-1412, SF-137 and SF-102). The probes were resuspended in sterile, nuclease-free water (Sigma Aldrich). Various cell lines were plated in 24-well plates to reach 70–80% confluency on the next day. Then the SmartFlare probes were diluted in sterile PBS (Lonza) and added to the cell cultures to a final concentration of 100 pM. The cells were incubated for 16–24 h and analysed using FACSCalibur flow cytometer (BD Bioscience). For experiments with fluorescently labelled dextrans, 293T cells were seeded in 96-well plates (2 × 10^4^ cells/well). Next day, fluorescein isothiocyanate- (FITC-) or rhodamine B isothiocyanate- (RITC-) dextran (average MW 10 kDa, Sigma-Aldrich) were added to the cells to a final concentration of 1 mg/ml. Following overnight incubation, the cells were washed 5 times with PBS and trypsinised. Equal amounts of FITC- and RITC-labelled cells were mixed and plated onto 96-well plate (2 × 10^4^ cells/well), then SmartFlare probes were added to a final concentration of 100 pM. After another overnight incubation the cells were harvested by trypsinisation and analysed using flow cytometer. All flow cytometry data were analysed with FlowJo v10.0.7 software (FlowJo LLC).

## Electronic supplementary material

Supplementary Information
