# Supplementary material for: SmartFlares fail to reflect their target transcripts levels
Source: Sci Rep. 2017 Sep 15;7:11682. doi: 10.1038/s41598-017-11067-6 (PMC5600982; doi:10.1038/s41598-017-11067-6)
Supplement: Supplementary file 1 — Supplementary Information [file 41598_2017_11067_MOESM1_ESM.pdf]

SmartFlares fail to reflect their target transcripts levels.

Maria Czarnek[1] and Joanna Bereta[1,*]

[1]Faculty of Biochemistry, Biophysics and Biotechnology, Jagiellonian University in Kraków, Gronostajowa 7, 30-387 Kraków, Poland

* joanna.bereta@uj.edu.pl

Supplementary Table S1. ΔCq values obtained in RT-qPCR assay. ΔCq was calculated as ΔCq = Cq (target gene) – Cq (reference gene). This number roughly reflects the difference in abundance between a target- and a reference transcript. In an idealized situation, at equal PCR efficiencies and similar amplicon lengths, ΔCq = 0 indicates equal concentrations of both transcripts; for reference/target transcript ratios equal 2, 10 and 100, ΔCq are respectively 1, 3.322 and 6.644.

Transcript/cells sample	ΔCq or ΔCq range	Figure[a]
HMOX1/293T		1a
WT	8.40	
KO clones	10.06 – 11.51	
WT/DMSO	8.45 – 8.91[b]	1d
WT/hemin	5.65 – 7.26[b]	
HMOX1/HeLa		1e
DMSO	8.53 – 8.91[b]	
hemin	2.38 – 3.91[b]	
IL6/HeLa		2a
ctrl	7.63	
stimul. IL1	2.98	
PTGS2/HeLa		2a
ctrl	7.14	
stimul. IL1	3.78	
IL6/U373-MG		2d
ctrl	6.99	
stimul. IL1	0.04	
PTGS2/U373-MG		2d
ctrl	n.d.	
stimul. IL1	11.43	
NRG1/MC38CEA		3a
WT	6.573	
NRG1[c]	1.67 – 2.32[c]	

[a] Figure in the main text; [b] the range for different times of cell incubation with DMSO or hemin; [c] the range for overexpression of NRG1 (I) or (III); n.d. – not detectable

Supplementary Table S2: Primers used for pX330-Pac-Cer vector construction (restriction enzymes recognition sites are in bold type; 2A peptide coding sequences are italicized)

Primer designation	Sequence
AgeI_SpCas9_For	**ACCGGT**GCCACCATGGACTATAA
T2A_SpCas9_Rev	*CAGACTTCCTCTGCCCTCTCCGCTTCC*CTTTTTCTTTTTTGCCTGGCC
T2A_Pac_For	*GGAAGTCTGCTAACATGCGGTGACGTCGAGGAGAATCCTGGACCT*ATGACCGAGTACAAGCCCAC
P2A_Pac_Rev	*GTCTCCAGCCTGCTTCAGCAGGCTGAAGTTAGTAGCTCCGCTTCC*GGCACCGGGCTTGCG
P2A_Cer_For	*AGCCTGCTGAAGCAGGCTGGAGACGTGGAGGAGAACCCTGGACCT*ATGGTGAGCAAGGGCGAG
EcoRI_Cer_Rev	**GAATTC**TTACTTGTACAGCTCGTCCATGC

Supplementary Table S3: Oligonucleotides used for cloning of HMOX1-targeting portion of sgRNA into pX330-Pac-Cer

sgRNA target site	Sequence	
HMOX-4	top	CACCGTGAAGCCGTCTCGGGTCACC
	bottom	AAACGGTGACCCGAGACGGCTTCAC
HMOX-21	top	CACCGCCTCCTTGGTGGCCTCCTTC
	bottom	AAACGAAGGAGGCCACCAAGGAGGC

Supplementary Table S4: Primers used for RT-qPCR and RT-PCR

Gene	Amplicon lenght [bp]	Forward primer	Reverse primer
hEF2	220	CGAGATCAAGGACAGTGTGG	AAGGTAGATGGGCTCCATGA
hHMOX1	165	TTCTTCACCTTCCCCAACATTG	CAGCTCCTGCAACTCCTCAAA
hIL6	148	TTCCAAAGATGTAGCCGCCC	TGCCTCTTTGCTGCTTTCAC
hPTGS2	177	ACCCACTCCAAACACAGTGC	GCTTCCCAGCTTTTGTAGCC
hERBB4	220	ACTGCTGCTTAACTGGTGTG	ACACTCCAGAGCCATCCATT
mEF2	149	CCACGGCAAGTCCACGCTGAC	AGAAGAGGGAGATGGCGGTGGATT
mHPRT	173	AGTCCCAGCGTCGTGATTAG	TGATGGCCTCCCATCTCCTT
mNrg1	170	ACCAGCCATCTCATAAAGTGTGC	AGATGCTTGTAGAAGCTGGCCATT

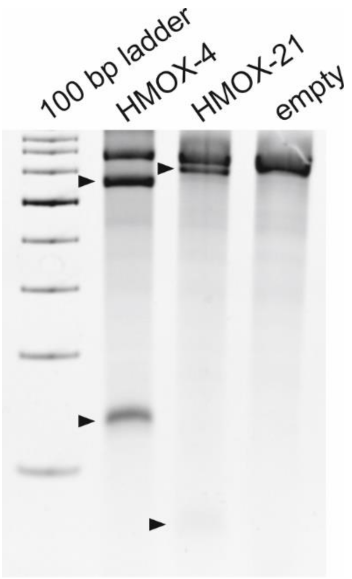

Supplementary Figure S1. CELI mismatch detection assay performed on DNA isolated from 293T transfected with one of two different *HMOX1*-targeting pX330-Pac-Cer vectors (HMOX-4 and HMOX-21, see Supplementary Table S2) or with control, empty vector (empty). Arrowheads indicate CELI digestion products.

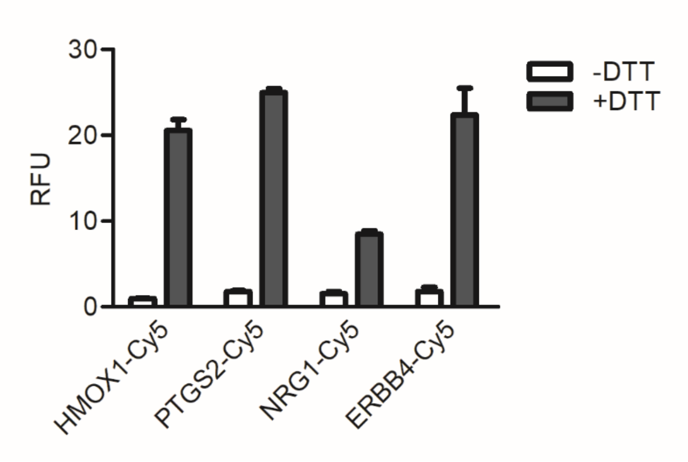

Supplementary Figure S2. Measurement of the fluorescence levels of 1 nM Cy5-labelled probes incubated for 5 min with 20 mM DTT or left untreated. Fluorescence of HMOX1-Cy5 without DTT is set as 1. Bars represent mean values with standard deviations of duplicates from a single experiment. The fluorescence was measured using Synergy H1 Hybrid Multi-Mode Microplate Reader (BioTek) (excitation was set at 630 nm and emission at 670 nm).

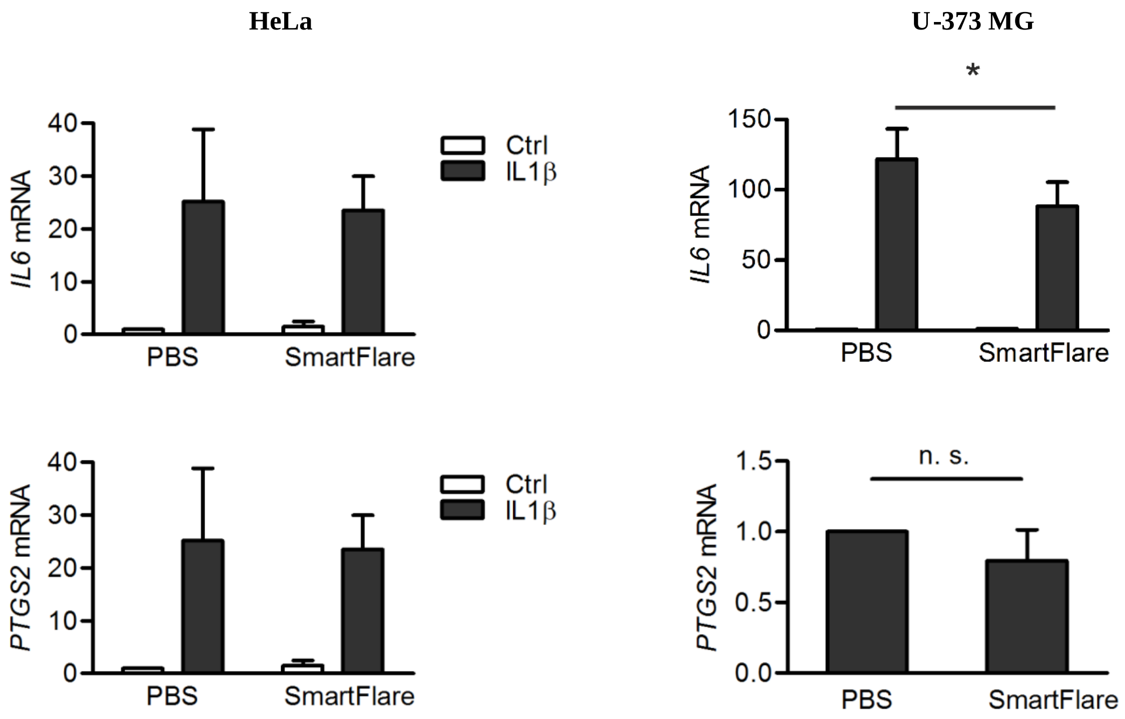

Supplementary Figure S3. RT-qPCR analysis of *IL6* and *PTGS2* expression in control and IL1β-stimulated HeLa and U-373 MG cells preincubated with 100 pM *PTGS2*-specific SmartFlare probe or PBS. Data shown as mean ± SEM from three independent experiments performed in duplicates. Because *PTGS2* mRNA is absent in unstimulated U-373 MG cells, *PTGS2* level in IL1β-stimulated cells preincubated with PBS is set as 1.

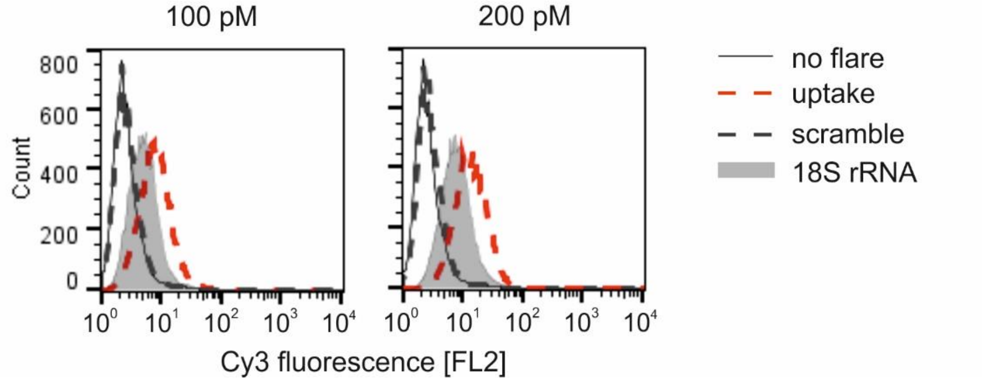

Supplementary Figure S4. Flow cytometry analysis of 293T cells incubated overnight with uptake-Cy3, scramble-Cy3 or 18S rRNA-Cy3 probes. Final concentrations of probes were 100 pM or 200 pM.

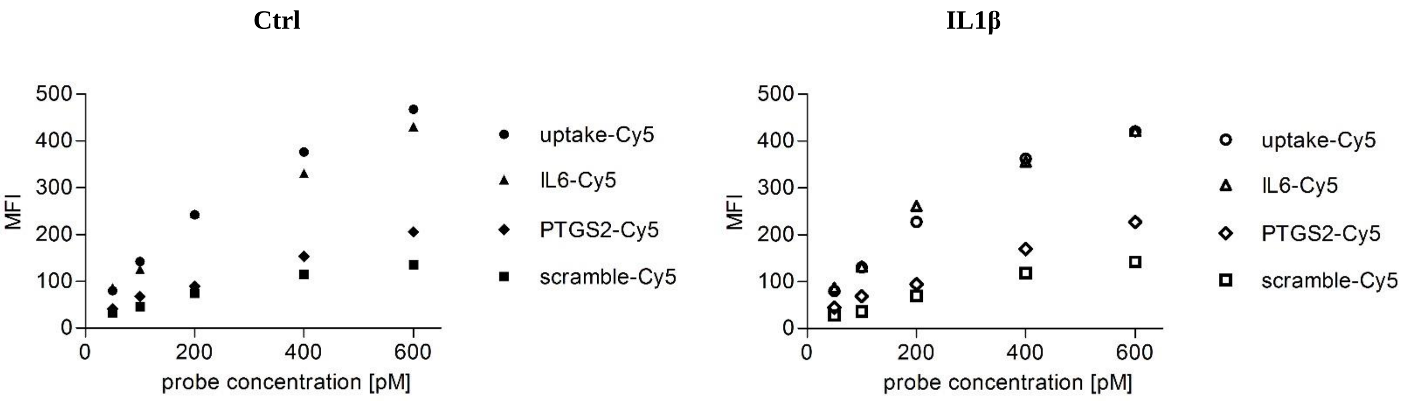

Supplementary Figure S5. MFI values of U-373 MG cells incubated overnight with uptake-Cy5, IL6-Cy5, PTGS2-Cy5 and scramble-Cy5 probes, then left untreated (Ctrl) or stimulated with 10 ng/ml IL1β for 3h. Final concentrations of probes were 50, 100, 200, 400 and 600 pM.

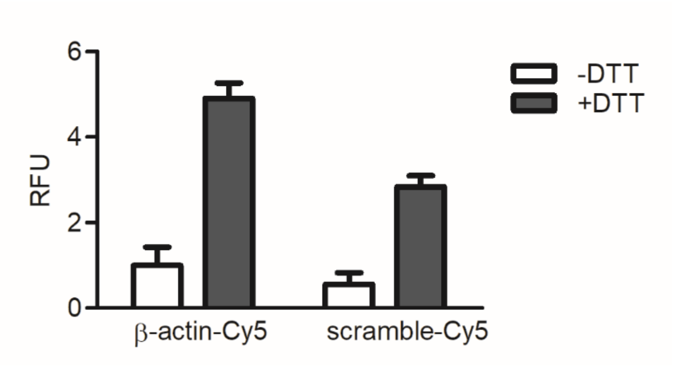

Supplementary Figure S6. Measurement of the fluorescences level of 1 nM Cy5-labelled probes, incubated for 5 min with 20 mM DTT or left untreated. Fluorescence of β-actin-Cy5 probe without DTT is set as 1. Bars represent mean values with standard deviations from three experiments performed in duplicates. The fluorescence was measured using Synergy H1 Hybrid Multi-Mode Microplate Reader (BioTek) (excitation was set at 630 nm and emission at 670 nm).

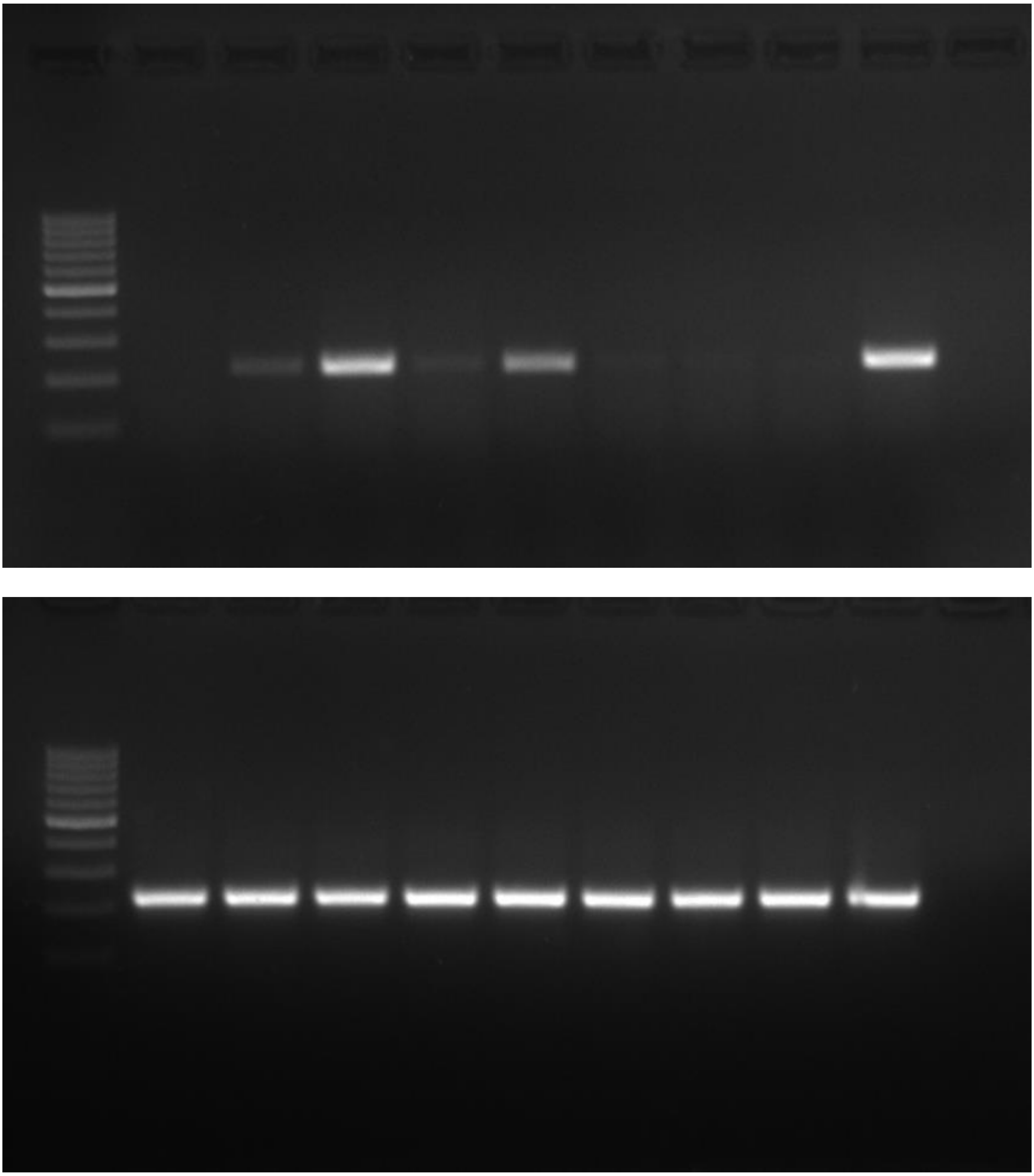

Supplementary Figure S7. The original unprocessed image from Figure 4 in the main text.

Supplementary Materials and Methods

Construction of pX330-Pac-Cer plasmid

Cas9 was PCR-amplified from pX330-U6-Chimeric_BB-Cbh-hSpCas9 with primers containing *Age*I site (forward) and a fragment of T2A peptide sequence (reverse). Pac (puromycin N-acetyltransferase) was PCR-amplified from pX260-U6-DR-BB-DR-Cbh-NLS-hSpCas9-NLS-H1-shorttracr-PGK-puro[1], (a gift from Feng Zhang, Addgene plasmid #422229) with primers containing fragments of T2A coding sequence (forward) and P2A coding sequence (reverse). Cerulean was amplified from LeGO-iCer2[2] (a gift from Boris Fehse, Addgene plasmid #27346) with primers containing a part of P2A coding sequence (forward), stop codon, and *Eco*RI restriction site (reverse). Primers were designed to introduce an overlap between T2A coding sequence in Cas9 and Pac, and an overlap between P2A in Pac and Cerulean coding sequences. PCR products were cleaned up from an agarose gel after electrophoresis and mixed. The whole Cas9-Pac-Cer cassette was PCR-amplified with *Age*I_SpCas9_For and *Eco*RI_Cer_Rev primers. PCR reactions were performed using Q5 polymerase (New England Biolabs). The resulting product (after subcloning step) was ligated into *Age*I/*Eco*RI-digested pX330-U6-Chimeric_BB-Cbh-hSpCas9.

CELI preparation

CELI nuclease was prepared as described by Till et al.[3]. Briefly, a bunch of fresh celery was juiced in a cold room, adjusted to 100 mM Tris-HCl, pH 7.7, 100 μM PMSF and centrifuged (2600×g, 20 min, 4ºC). The supernatant was brought to 25% saturation of $(NH_4)_2SO_4$, mixed for 30 min at 4°C and centrifuged (16 000 g, 40 min, 4ºC). The supernatant was then adjusted to 77% saturation of $(NH_4)_2SO_4$, mixed for 30 min at 4°C and centrifuged (16 000 g, 90 min, 4ºC). The pellet containing salted-out CELI nuclease was suspended in 1/10 of the starting volume of 100 mM Tris-HCl, pH 7.7, 500 mM KCl, 1 mM PMSF. The solution was dialysed overnight against 12 l of 100 mM Tris-HCl, pH 7.7, 500 mM KCl, 1 mM PMSF with two buffer changes. The resulting solution is

regarded as CELI nuclease.

CELI mismatch detection assay

The region in genomic DNA surrounding the CRISPR target sites of *HMOX1* was PCR amplified (forward primer: AGCCAGCTTTGTGTTCACCT, reverse primer: GAGGCACCCTCAGTCTCA CT). PCR products (10 µl) were subjected to heteroduplex formation (5 µl of reaction with DNA from the original cell line + 5 µl of reaction with DNA from a *HMOX1*-targeted clone) with the following program: 95ºC for 10 min, 95ºC to 85ºC cooling at −2ºC/s, 85ºC to 25ºC at −0.25ºC/s, followed by incubation at 25ºC for 1 min. DNA was subjected to digestion with mismatch-specific CELI nuclease (0.5 µl per sample) and 1.5 µl of 10×CELI reaction buffer (100 mM HEPES pH 7.5, 100 mM $MgSO_4$, 200 mM KCl, 0.2% Triton X-100, 2 µg/ml BSA) in a 15 µl total reaction volume for 45 min at 45°C. The cleavage products were resolved in 6% acrylamide gel in TBE buffer following staining in TBE buffer containing SERVA DNA Stain G (DNA from cell populations) or in 2% agarose gel containing SERVA DNA Stain G in TAE buffer (DNA from cell clones). Bands were visualized with gel imaging system Quantum ST5 (Vilber Lourmat).

Supplementary references

1. Cong, L. *et al.* Multiplex genome engineering using CRISPR/Cas systems. *Science* **339,** 819–823 (2013).
2. Weber, K., Bartsch, U., Stocking, C. & Fehse, B. A multicolor panel of novel lentiviral ‘gene ontology’ (LeGO) vectors for functional gene analysis. *Mol. Ther. J. Am. Soc. Gene Ther.* **16,** 698–706 (2008).
3. Till, B. J., Zerr, T., Comai, L. & Henikoff, S. A protocol for TILLING and Ecotilling in plants and animals. *Nat. Protoc.* **1,** 2465–2477 (2006).
